# Maize GOLDEN2-LIKE proteins enhance drought tolerance in rice by promoting stomatal closure

**DOI:** 10.1093/plphys/kiad561

**Published:** 2023-10-18

**Authors:** Xia Li, Jing Li, Shaobo Wei, Yuan Gao, Hongcui Pei, Rudan Geng, Zefu Lu, Peng Wang, Wenbin Zhou

**Affiliations:** Institute of Crop Sciences, Chinese Academy of Agricultural Sciences, Beijing 100081, China; Institute of Crop Sciences, Chinese Academy of Agricultural Sciences, Beijing 100081, China; Institute of Crop Sciences, Chinese Academy of Agricultural Sciences, Beijing 100081, China; Institute of Crop Sciences, Chinese Academy of Agricultural Sciences, Beijing 100081, China; Institute of Crop Sciences, Chinese Academy of Agricultural Sciences, Beijing 100081, China; Institute of Crop Sciences, Chinese Academy of Agricultural Sciences, Beijing 100081, China; Institute of Crop Sciences, Chinese Academy of Agricultural Sciences, Beijing 100081, China; CAS Center for Excellence in Molecular Plant Sciences, Institute of Plant Physiology and Ecology, Chinese Academy of Sciences, Shanghai 200032, China; Institute of Crop Sciences, Chinese Academy of Agricultural Sciences, Beijing 100081, China

## Abstract

Drought has become one of the most severe abiotic stresses experienced in agricultural production across the world. Plants respond to water deficit via stomatal movements in the leaves, which are mainly regulated by abscisic acid (ABA). A previous study from our lab showed that constitutive expression of maize (*Zea mays* L.) GOLDEN2-LIKE (GLK) transcription factors in rice (*Oryza sativa* L.) can improve stomatal conductance and plant photosynthetic capacity under field conditions. In the present study, we uncovered a function of ZmGLK regulation of stomatal movement in rice during drought stress. We found that elevated drought tolerance in rice plants overexpressing *ZmGLK1* or *GOLDEN2* (*ZmG2*) was conferred by rapid ABA-mediated stomatal closure. Comparative analysis of RNA-sequencing (RNA-seq) data from the rice leaves and DNA affinity purification sequencing (DAP-seq) results obtained in vitro revealed that ZmGLKs played roles in regulating ABA-related and stress-responsive pathways. Four upregulated genes closely functioning in abiotic stress tolerance with strong binding peaks in the DAP-seq data were identified as putative target genes of ZmGLK1 and ZmG2 in rice. These results demonstrated that maize GLKs play an important role in regulating stomatal movements to coordinate photosynthesis and stress tolerance. This trait is a valuable target for breeding drought-tolerant crop plants without compromising photosynthetic capacity.

## Teaser

Maize GOLDEN2-LIKE proteins confer drought tolerance in rice by promoting abscisic acid–mediated stomatal closure and preventing water loss.

## Introduction

Global crop production must be approximately doubled by 2050 to meet the demands of the increasing human population (FAO 2009; Tilman et al. 2011). However, yield improvement has stagnated in recent years and is clearly projected to fall short of the expected demand (Ray et al. 2013). Yield stagnation in major crops is caused by a combination of factors including climate change, soil erosion, and cultivar restriction (Ray et al. 2012). Drought is one of the most severe natural hazards in agricultural production; it has affected large agricultural areas and been exacerbated worldwide over the last 40 yr (FAO 2021). Rice (*Oryza sativa* L.) serves as a staple food for nearly half of the world's population, but it is a high water-consuming crop and is particularly susceptible to drought. Over 50% of the world's rice production is estimated to be affected by drought stress (Ambavaram et al. 2014). Development of low-water-consuming and drought-tolerant rice varieties is urgently needed to meet global food demand under changing climatic conditions (Todaka et al. 2012).

Stomata are the main channels for the gas exchange between plant and the atmosphere. When plants suffer from water deficit or are exposed to other environmental stimuli (e.g. low light intensity, low air humidity, high CO_2_ levels, and pathogens), stomata are rapidly closed, especially in angiosperms (Sierla et al. 2018). This dynamic movement is driven by turgor pressure changes in the guard cells, as a result of the activation of anion channels and the inhibition of inward-rectifying K^+^ channels, which encoded by *K^+^ CHANNEL IN ARABIDOPSIS THALIANA* (*KAT*) and *ARABIDOPSIS K^+^ TRANSPORTER* (*AKT*) genes (Kim et al. 2010). The efflux of anions and small metabolites, including Cl^−^, NO_3_^−^, and malate, causes membrane depolarization to activate the outward-rectifying K^+^ channel and facilitates K^+^ efflux, further reducing turgor pressure inside the guard cells and leading to the stomatal closure (Pandey et al. 2007). Under water-deficit conditions, the phytohormone abscisic acid (ABA) plays as the primary regulator of stomatal movement to prevent water loss, in which endogenous ABA levels are controlled by a precise balance between biosynthesis and catabolism, which also influenced by transport and conjugation process (Kushiro et al. 2004; Hsu et al. 2021). ABA is initially synthesized from C_40_ carotenoids to form xanthophylls (e.g. 9-*cis*-violaxanthin and 9-*cis*-neoxanthin); a C_15_ intermediate, xanthoxin, is formed in the plastids via oxidative cleavage catalyzed by 9-*cis*-epoxycarotenoid dioxygenase (NCED). Xanthoxin is then exported to the cytosol and converted to ABA through a 2-step reaction via short-chain dehydrogenase/reductase 1 (SDR1/ABA2) and Arabidopsis aldehyde oxidase 3 (AAO3) (Seo and Koshiba 2002; Xiong and Zhu 2003).

Transcription factors (TFs) are crucial regulators of many biological processes, including responses to environmental signals and hormone regulation. These regulatory functions are accomplished through binding to specific *cis*-elements in the promoter regions of target genes (Todaka et al. 2012). Numerous abiotic stress-responsive TFs have been identified in plants; for instance, WRKY, MYB, and DREB/CBF TFs have all been reported as key regulators of plant stress responses (Manna et al. 2021). GOLDEN2-LIKE (GLK) TFs generally act as transcriptional activators of chloroplast development and biogenesis (Rossini et al. 2001; Wang et al. 2013) and play important roles in regulating nuclear photosynthesis-related genes (Chen et al. 2016). In maize (*Zea mays* L.), 2 *GLK* genes, *ZmGLK1* and *GOLDEN2* (*ZmG2*), have shown differential expression patterns between mesophyll cells and the bundle sheath (Hall et al. 1998; Chang et al. 2012). Ectopic overexpression of maize *GLK* genes in rice induces chloroplast development in bundle sheath cells and activates intracellular plasmodesmatal connections, considering the key step in forming intermediate proto-Kranz anatomy in the transition from C_3_ to C_4_ photosynthesis (Wang et al. 2017). A previous study from our lab showed that constitutive *ZmGLK* expression in rice leads to increased xanthophyll content and further mitigates the photoinhibition under high-light conditions, resulting in an enhanced photosynthetic capacity with higher stomatal conductance and improved biomass and grain yield in the field (Li et al. 2020). Moreover, GLKs also function in abiotic stress responses (Ahmad et al. 2019) and pathogen resistance (Murmu et al. 2014); for example, GLKs affect stomatal movement in Arabidopsis (*Arabidopsis thaliana*) when exposed to ozone (Nagatoshi et al. 2016).

In this study, we uncovered the dual function of maize GLKs, and that ectopic overexpression of *ZmGLK1* and *ZmG2* in rice conferred improved drought tolerance by promoting stomatal closure in response to water deficit while maintaining high stomatal conductance to obtain efficient photosynthesis when sufficient water was available. We further showed that rapid stomatal movement was mediated by ABA-involved pathway under drought conditions. These results suggest that *GLK* genes may be promising candidates for breeding rice varieties with high stomata flexibility and sustainable yield, which would strongly improve agricultural production and increase food security in the context of climate change.

## Results

### ZmGLK1 and ZmG2 conferred improved drought tolerance in rice

**Figure 1.**
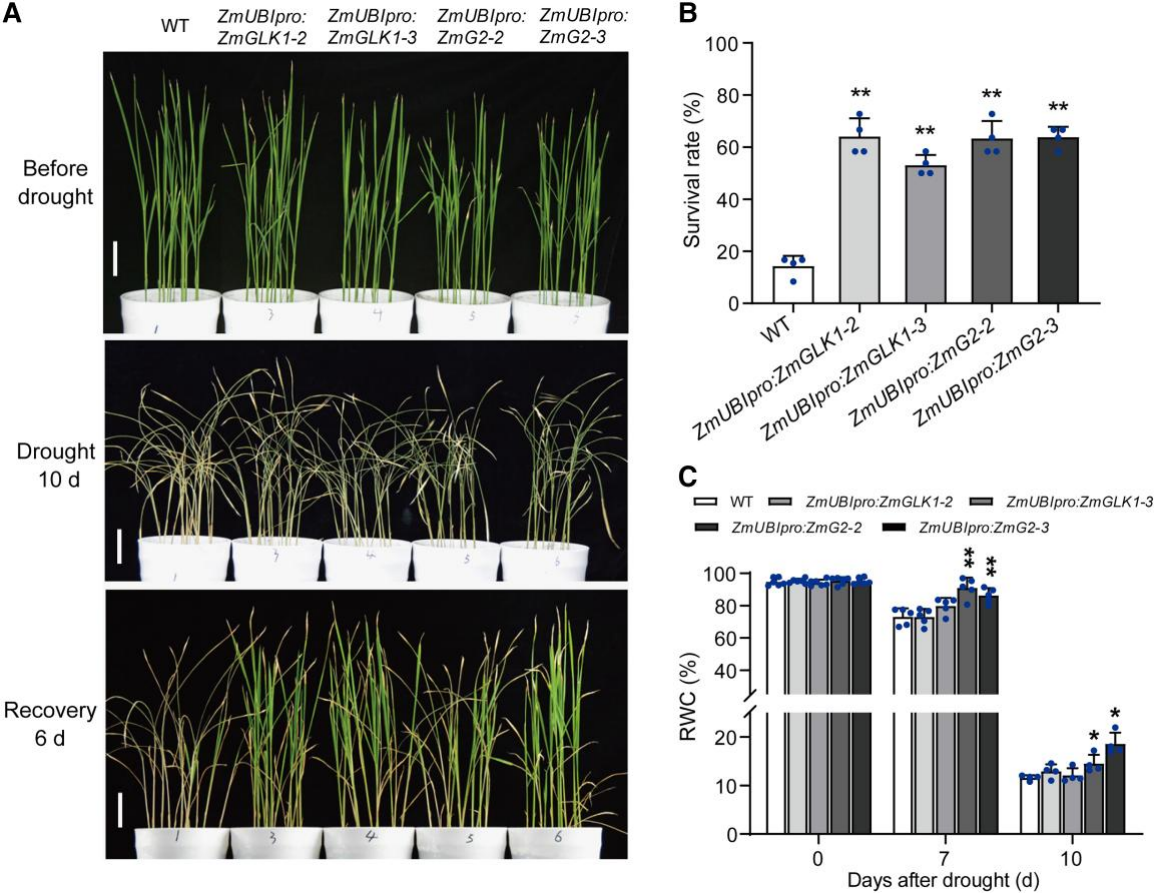
Overexpression of *ZmGLK1* and *ZmG2* in rice increased drought tolerance. **A)** Phenotypes of WT, *ZmUBI_pro_*:*ZmGLK1*, and *ZmUBI_pro_*:*ZmG2* rice plants during drought stress. Three-week-old WT, *ZmUBI_pro_*:*ZmGLK1*, and *ZmUBI_pro_*:*ZmG2* rice seedlings grown in soil were drought stressed by withholding water for 10 d and then watered for a 6-d recovery period. The upper, middle, and lower panels show representative plants before drought stress, after 10 d of drought stress, and after the 6-d recovery, respectively. Scale bar: 2 cm. **B)** Survival rates of WT, *ZmUBI_pro_*:*ZmGLK1*, and *ZmUBI_pro_*:*ZmG2* rice plants after 10 d of drought stress followed by 6 d of recovery. Data are presented as the mean ± Sd from 4 biological replicates. **C)** The RWC of WT, *ZmUBI_pro_*:*ZmGLK1*, and *ZmUBI_pro_*:*ZmG2* rice leaves after 0, 7, and 10 d of drought stress. Data are presented as the mean ± Sd from 4 to 6 biological replicates. *P < 0.05, **P < 0.01 (Student's *t* test).

In our previous study, field-grown transgenic rice lines constitutively expressing *ZmGLK1* or *ZmG2* driven by the maize *Ubiquitin* (*ZmUBI*) promoter performed improved photosynthesis rates and higher stomatal conductance (Li et al. 2020). We further explored the stomatal responses of transgenic rice plants to water deficit with pot experiments in the growth chamber. Surprisingly, transgenic rice plants exhibited stronger drought tolerance than wild-type (WT) plants after recovery from a 10-d drought treatment (Fig. 1A). Specifically, the survival rates of *ZmUBI_pro_*:*ZmGLK1* and *ZmUBI_pro_*:*ZmG2* plants were 53.0% to 64.0% after the 6-d recovery period, which were significantly higher than the WT (14.3%; Fig. 1B). Moreover, the relative water content (RWC) in the leaves of WT and transgenic plants ranged from 94.7% to 95.3% before drought but decreased to 73.1% in the WT after water was withheld for 7 d. In comparison, *ZmUBI_pro_*:*ZmGLK1* and *ZmUBI_pro_*:*ZmG2* plants maintained a relatively high RWC, especially *ZmUBI_pro_*:*ZmG2*, ranging from 86.2% to 90.9%. After 10 d of drought stress, the RWC values of WT and *ZmUBI_pro_*:*ZmGLK1* plants decreased to 11.6% to 12.9%, which were significantly lower than those of *ZmUBI_pro_*:*ZmG2* plants (14.5% to 18.6%; Fig. 1C). These results indicated ZmGLK1 and ZmG2 both conferred higher capacities for water conservation and thus drought tolerance.

We next tested the growth performance of WT, *ZmUBI_pro_*:*ZmGLK1*, and *ZmUBI_pro_*:*ZmG2* rice plants to PEG-induced osmotic stress as a drought simulation. After growth in 20% PEG 6000 for 10 d, *ZmUBI_pro_*:*ZmGLK1* and *ZmUBI_pro_*:*ZmG2* rice plants showed less wilting and chlorosis compared to the WT (Supplemental Fig. S1A). The maximum quantum efficiency of photosystem II (PSII; *F*_v_/*F*_m_) was measured as an important indicator of plant physiological state under stress conditions, and that the *F*_v_/*F*_m_ values were significantly higher in *ZmUBI_pro_*:*ZmGLK1* and *ZmUBI_pro_*:*ZmG2* rice plants (0.793 and 0.803, respectively) than in the WT (0.765) after 10 d of PEG treatment (Supplemental Fig. S1B). We also monitored changes of RWC in rice seedling during PEG treatment. The results showed that the transgenic plants retained significantly higher RWC compared to the WT. Specifically, RWC values were 11.4% to 12.1% and 29.5% to 29.7% higher in *ZmUBI_pro_*:*ZmGLK1* and *ZmUBI_pro_*:*ZmG2* rice plants, respectively, compared with the WT (Supplemental Fig. S1C). These results together indicated that overexpression of *ZmGLK1* and *ZmG2* in rice significantly improve the tolerance to drought and osmotic stress.

### ZmGLK1 and ZmG2 triggered rapid stomatal closure in drought-stressed rice plants

To further investigate the physiological mechanism underlying the elevated drought tolerance conferred by ZmGLK1 and ZmG2, we evaluated the effects of drought treatment on stomatal traits of rice seedlings grown in the pots in the growth chamber, since stomata are the main channels for gas exchange and water respiration in plants, serving as the dominant limitation to photosynthesis under drought. We therefore first measured stomatal conductance and photosynthetic-related parameters under control conditions using a LICOR-6400XT portable photosynthesis system. The results revealed significantly higher stomatal conductance in *ZmUBI_pro_*:*ZmGLK1* and *ZmUBI_pro_*:*ZmG2* rice seedlings (0.118–0.139 and 0.126–0.131, respectively) compared with the WT (0.083) under control condition; while the transgenic plants also performed higher photosynthesis rates, intercellular CO_2_ concentrations (Ci), and transpiration rates (Supplemental Fig. S2), as the plants grown in the field (Li et al. 2020). In contrast, after 7 d of drought treatment, *ZmUBI_pro_*:*ZmGLK1* and *ZmUBI_pro_*:*ZmG2* rice plants displayed sharply decrease in stomatal conductance (0.062–0.073 and 0.054–0.059, respectively), whereas that of WT remained relatively stable under drought conditions (0.087; Supplemental Fig. S2B). The photosynthesis rates, C*i*, and transpiration rates showed corresponding declines in *ZmUBI_pro_*:*ZmGLK1* and *ZmUBI_pro_*:*ZmG2* rice plants during water deprivation (Supplemental Fig. S2, A, C, and D).

**Figure 2.**
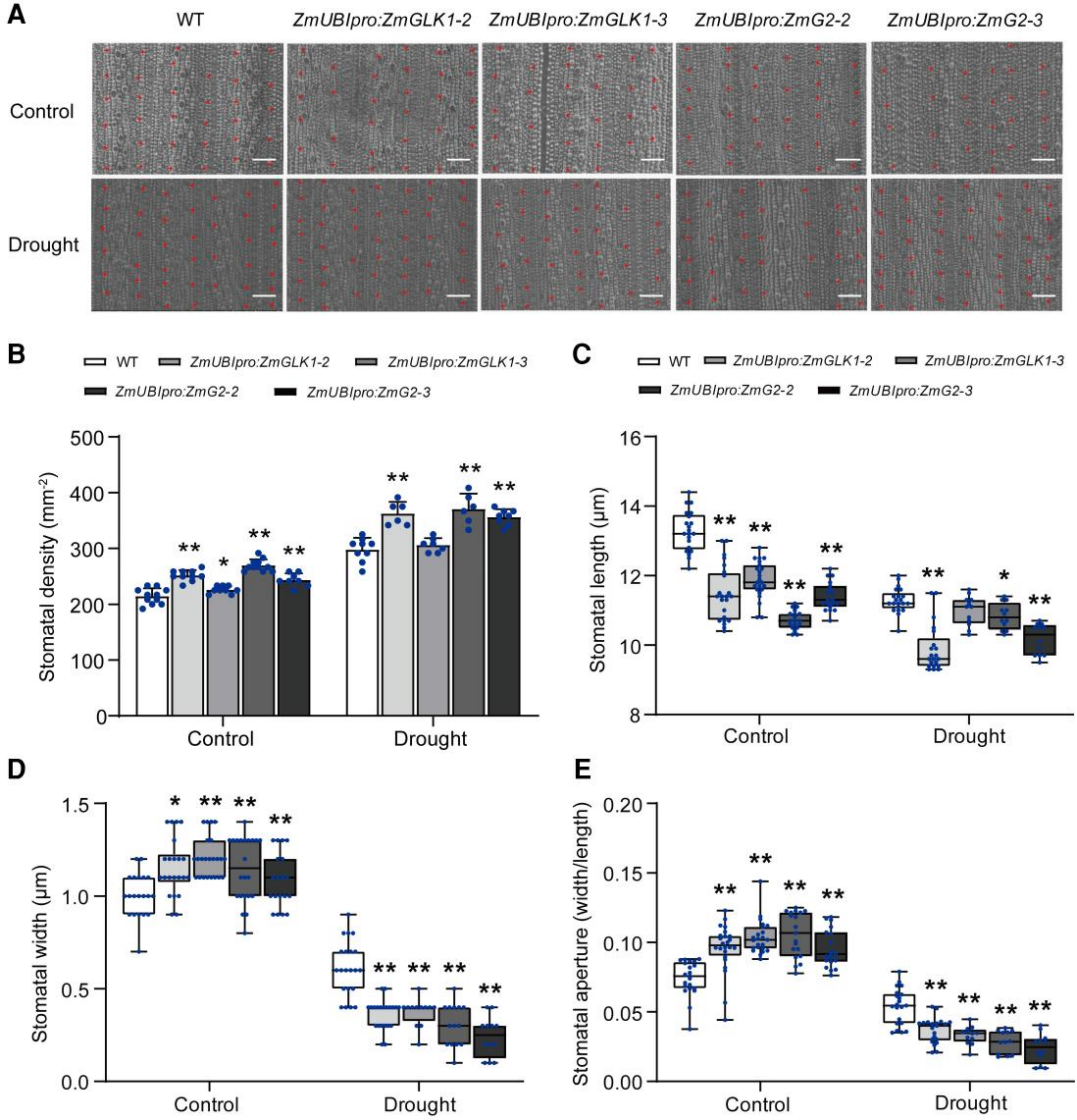
Comparison of stomatal density and stomatal opening status in WT, *ZmUBI_pro_:ZmGLK1*, and *ZmUBI_pro_*:*ZmG2* rice plants under normal growth conditions or drought stress for 7 d. **A)** Scanning electron microscope (SEM) images of stomata in WT, *ZmUBI_pro_*:*ZmGLK1*, and *ZmUBI_pro_*:*ZmG2* rice leaves. Scale bar: 50 *μ*m. **B)** Stomatal density, **C)** stomatal length, **D)** stomatal aperture, and **E)** aperture ratio in leaves from 3-wk-old WT, *ZmUBI_pro_*:*ZmGLK1*, and *ZmUBI_pro_*:*ZmG2* plants grown in soil under normal growth conditions or drought stress for 7 d. Data in **B)** are shown as the mean ± Sd (*n* > 8 biological replicates). Box plots in **C to E)** shows median (horizontal line) and individual values (dots; *n* > 15 biological replicates). *P < 0.05, **P < 0.01 (Student's *t* test).

We next compared the stomatal traits between WT and *ZmUBI_pro_*:*ZmGLK1* or *ZmUBI_pro_*:*ZmG2* rice plants under both control and drought conditions. Transgenic plants presented higher stomatal density in the leaves but had significantly shorter stomata compared to the WT regardless of conditions (Fig. 2, A to C). Intriguingly, the stomata were prominently wider in *ZmUBI_pro_*:*ZmGLK1* and *ZmUBI_pro_*:*ZmG2* rice leaves compared to the WT under control conditions (Fig. 2D), whereas under drought stress, the stomatal widths were significantly decreased in transgenic plants to a lower level than WT, consistent with the stomatal aperture data (Fig. 2E).

**Figure 3.**
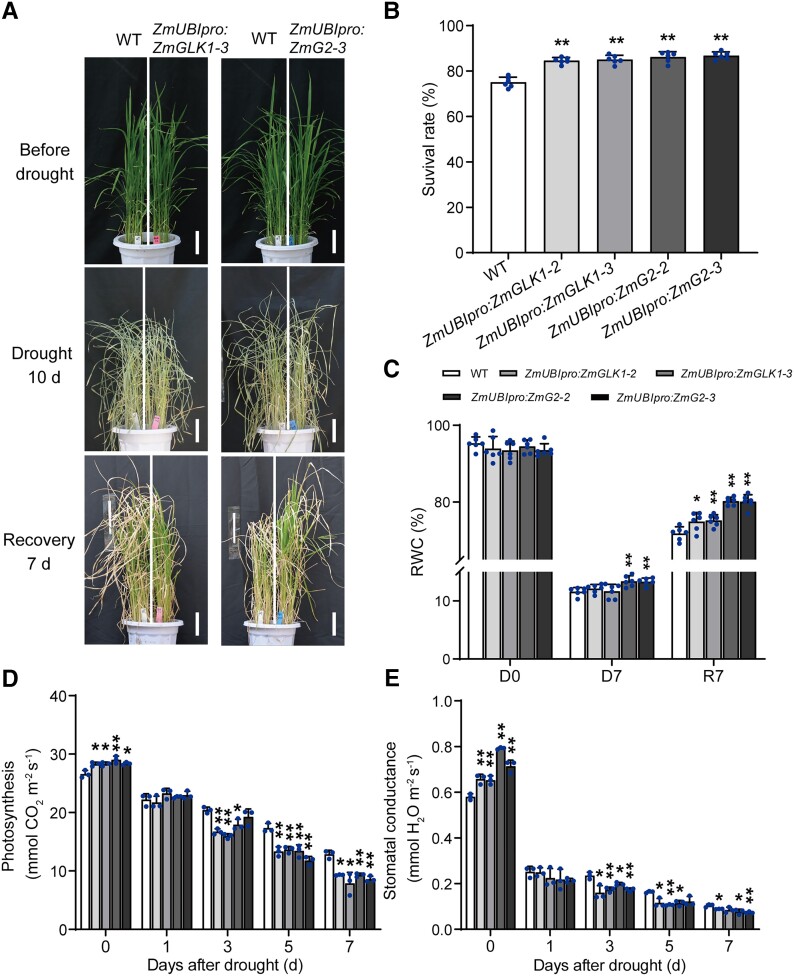
ZmGLKs conferred rapid stomatal closure to prevent water loss in rice during drought. **A)** Phenotypes of WT, *ZmUBI_pro_*:*ZmGLK1*, and *ZmUBI_pro_*:*ZmG2* rice plants during drought stress. Sixty-day-old WT, *ZmUBI_pro_*:*ZmGLK1*, and *ZmUBI_pro_*:*ZmG2* rice plants grown in soil in the greenhouse with natural light were drought stressed by withholding water for 10 d and then rewatered for a 7-d recovery period. The upper, middle, and lower panels show representative plants before drought stress, after 10 d of drought stress, and after the 7-d recovery, respectively. Scale bar: 10 cm. **B)** Survival rates of WT, *ZmUBI_pro_*:*ZmGLK1*, and *ZmUBI_pro_*:*ZmG2* rice plants after 10 d of drought stress followed by 7 d of recovery. **C)** The RWC of WT, *ZmUBI_pro_*:*ZmGLK1*, and *ZmUBI_pro_*:*ZmG2* rice leaves after 0 and 7 d of drought stress and after 7 d of recovery. **D, E)** Dynamic change of photosynthesis rate **D)** and stomatal conductance **E)** of WT, *ZmUBI_pro_*:*ZmGLK1*, and *ZmUBI_pro_*:*ZmG2* rice plants during the drought stress. Data are presented as the mean ± Sd from 3 to 6 biological replicates. *P < 0.05, **P < 0.01 (Student's *t* test).

Considering the relative low light intensity in the growth chamber could lead to the stomatal closure, we further conducted a pot experiment in the greenhouse with natural light to exclude the influence of low light. As expected, the results showed consistency with the chamber experiment (Fig. 1). All plants were severely impaired due to the rapid loss of water, during the 10-d drought duration (Supplemental Fig. S3; Fig. 3A). After rewatering for 7 d, we observed the higher survival rate in *ZmUBI_pro_*:*ZmGLK1* and *ZmUBI_pro_*:*ZmG2* rice plants (Fig. 3B), as well as the significantly higher RWC of leaves than WT either during the drought or the recovery stage (Fig. 3C). Moreover, we monitored the dynamics of photosynthesis rate and stomatal conductance throughout the duration of drought, and that *ZmUBI_pro_*:*ZmGLK1* and *ZmUBI_pro_*:*ZmG2* rice plants performed higher photosynthesis rate and stomatal conductance under sufficient water condition. Nevertheless, the photosynthesis rate and stomatal conductance of all plants were generally declined as the drought deepened, of which *ZmUBI_pro_*:*ZmGLK1* and *ZmUBI_pro_*:*ZmG2* rice plants presented lower photosynthesis rate and the stomatal conductance compared to the WT (Fig. 3, D and E). These results together clearly indicated that the rapid stomata closure was triggered by water deficiency in *ZmUBI_pro_*:*ZmGLK1* and *ZmUBI_pro_*:*ZmG2* rice plants, further contributing to the elevated drought tolerance.

### Regulation of rapid stomatal closure was ABA mediated in *ZmUBI_pro_*:*ZmGLK1* and *ZmUBI_pro_*:*ZmG2* rice plants

**Figure 4.**
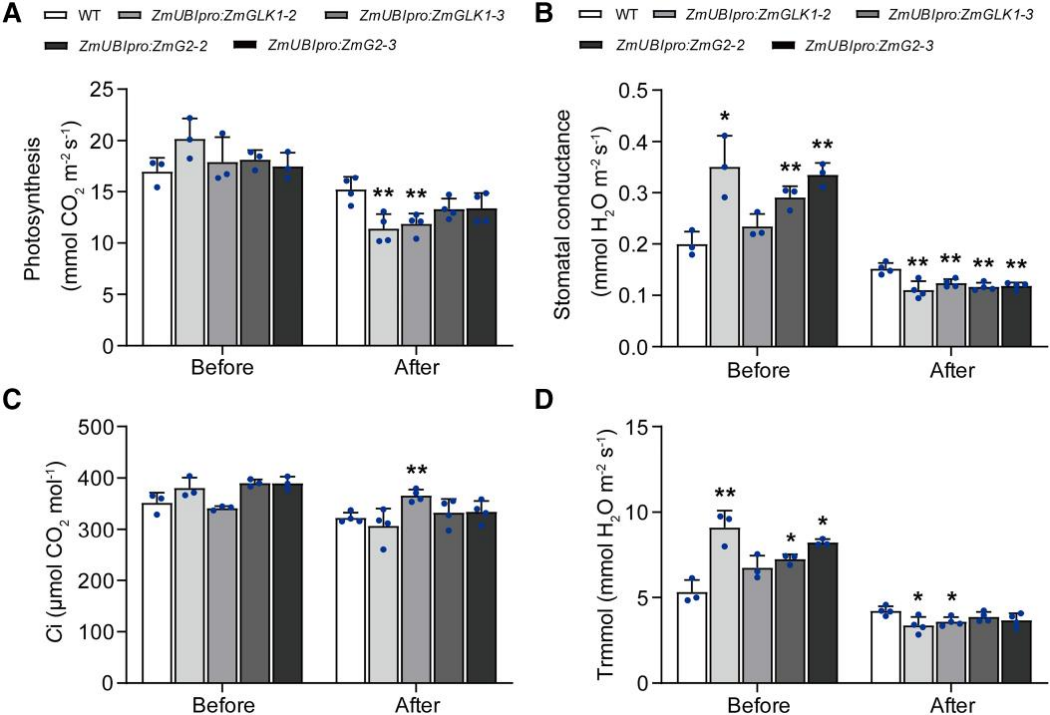
Exogenous ABA application reduced the photosynthesis rate and stomatal conductance in rice plants overexpressing *ZmGLK1* or *ZmG2* compared to the WT. **A)** Photosynthesis rates, **B)** stomatal conductance, **C)** Ci, and **D)** transpiration rates of 3-wk-old WT, *ZmUBI_pro_*:*ZmGLK1*, and *ZmUBI_pro_*:*ZmG2* rice plants grown in soil before or 2.5 h after ABA treatment. Data are shown as the mean ± Sd from 3 biological replicates. *P < 0.05, **P < 0.01 (Student's *t* test).

During the drought stress, ABA is the pivotal phytohormone that regulates stomatal movement to respond drought (Chen et al. 2020). To further dissect the underlying mechanism associated with stomatal movement induced by *ZmGLK1* and *ZmG2*, we treated rice plants with ABA to clarify whether the rapid stomatal closure was ABA induced. After 2.5 h of applying 100 *μ*M ABA, *ZmUBI_pro_*:*ZmGLK1* and *ZmUBI_pro_*:*ZmG2* rice plants showed strongly decreased photosynthesis rates, accompanied with the reduced stomatal conductance (Fig. 4, A and B). Accordingly, the C*i* and transpiration rate were generally lower in *ZmUBI_pro_*:*ZmGLK1* and *ZmUBI_pro_*:*ZmG2* rice plants compared with the WT after ABA application (Fig. 4, C and D). The effects of exogenous ABA application on photosynthetic traits and stomatal conductance in the WT and transgenic plants mimicked the results obtained from the drought stress treatments, which indicated the regulation of rapid stomatal closure in response to water-deficit stress conferred by ZmGLK1 and ZmG2 was ABA mediated.

### ZmGLK1 and ZmG2 regulated stomata-related genes to promote drought tolerance

**Figure 5.**
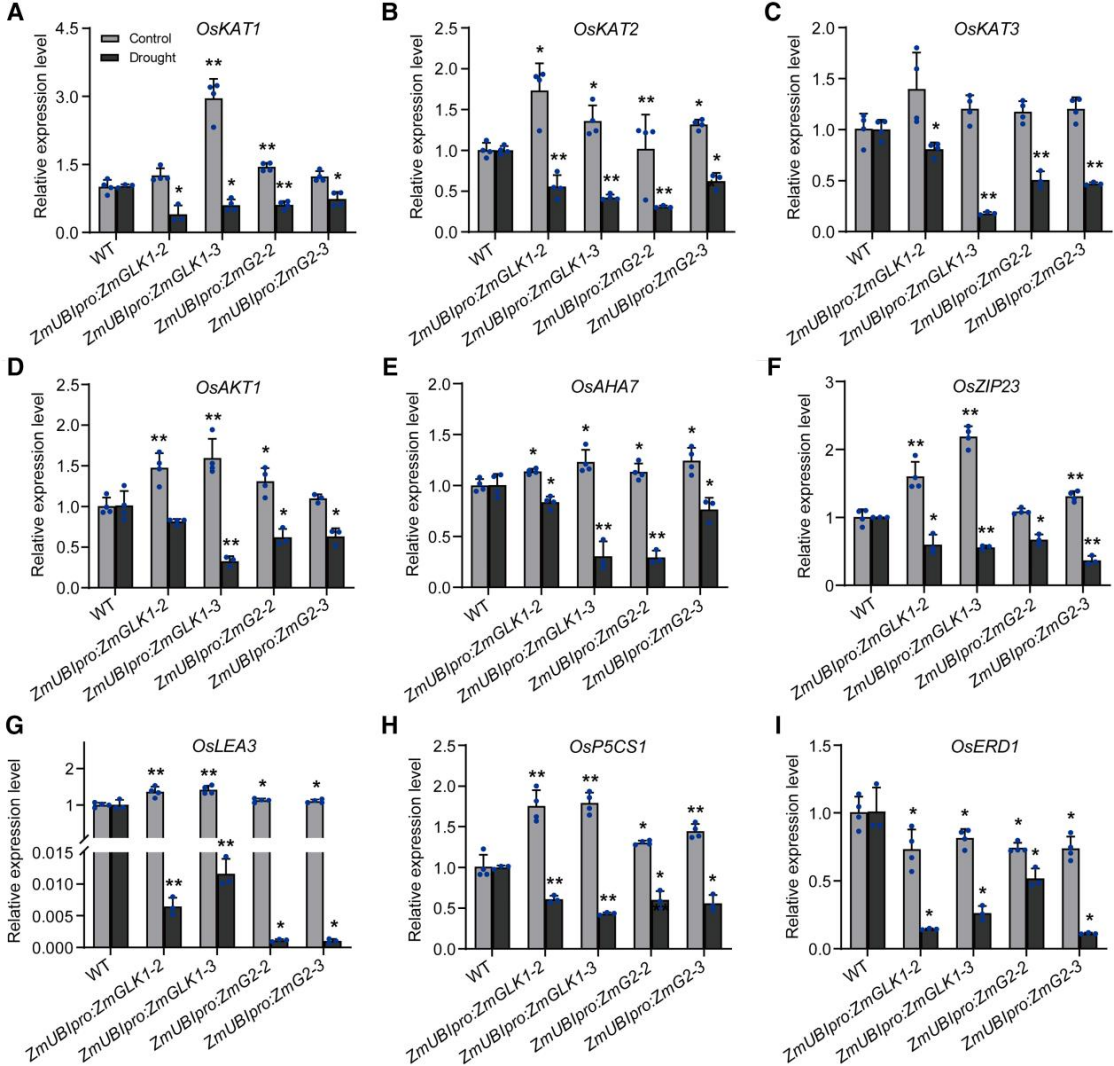
Relative expression levels of genes involved in stomatal movement and stomatal aperture in WT, *ZmUBI_pro_*:*ZmGLK1*, and *ZmUBI_pro_*:*ZmG2* rice under normal conditions and after 7 d of drought stress. Expression levels of **A)***OsKAT1*, **B)***OsKAT2*, **C)***OsKAT3*, **D)***OsAKT1*, **E)***OsAHA7*, **F)***OsZIP23*, **G)***OsLEA3*, **H)***OsP5CS1*, and **I)***OsERD1*. Gene expression levels were measured with RT-qPCR in the leaves of 3-wk-old rice plants grown in soil under normal conditions or drought stress for 7 d. Data are presented as the mean ± Sd from 3 biological replicates. *P < 0.05, **P < 0.01 (Student's *t* test).

To further understand the molecular mechanisms regulated by ZmGLKs under drought stress, we next compared the expression levels of several genes associated with stomatal movement in WT, *ZmUBI_pro_*:*ZmGLK1*, and *ZmUBI_pro_*:*ZmG2* rice plants under control and drought stress conditions. Under control conditions, several key genes were highly expressed in the transgenic plants compared with the WT but profoundly downregulated in response to drought stress. These comprised 4 genes encoding proteins associated with inward rectifying shaker-like potassium channels (3 *OsKAT*s and 1 *OsAKT1* gene), 1 H^+^-ATPase (*OsAHA7*), and several stress-responsive genes (including *OsbZIP23*, *OsP5CS1*, and *OsLEA3*; Fig. 5). These results demonstrated that ZmGLK1 and ZmG2 improved drought tolerance by downregulating genes involved in stomatal movement when suffering from water deficit.

**Figure 6.**
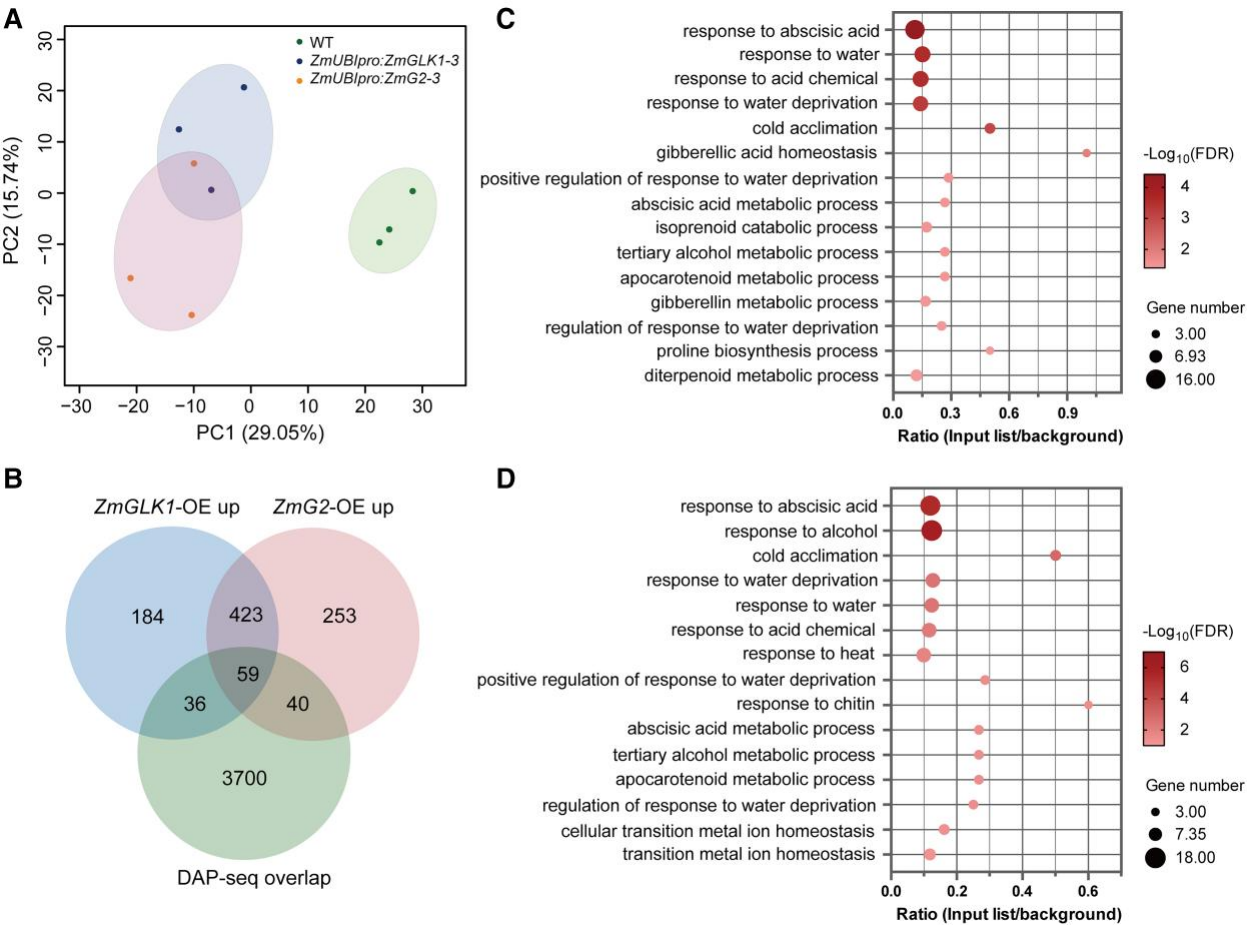
Transcriptomic analysis of WT, *ZmUBI_pro_:ZmGLK1*, and *ZmUBI_pro_*:*ZmG2* rice plants at 3 h after ABA treatment. **A)** PCA of gene expression in WT, *ZmUBI_pro_*:*ZmGLK1-3*, and *ZmUBI_pro_*:*ZmG2-3* rice plants based on RNA-seq data. **B)** Unique and overlapping DEGs upregulated in *ZmUBI_pro_*:*ZmGLK1* and *ZmUBI_pro_*:*ZmG2* rice plants compared to the WT and unique and overlapping *ZmGLK1* and *ZmG2* target genes identified from DAP-seq data. DEGs were identified based on |log_2_ (fold change)| > 1 and *P* < 0.05 by “DESeq” R package. **C, D)** GO functional categories for DEGs upregulated in *ZmUBI_pro_*:*ZmGLK1***C)** and *ZmUBI_pro_*:*ZmG2***D)** rice plants compared to the WT. Bubble size indicates the number of DEG counts in the corresponding GO category; bubble intensity corresponds to the −log_10_(false discovery rate [FDR] value); and the *x*-axis indicates the ratio of DEGs in each GO category to all genes in the category.

**Figure 7.**
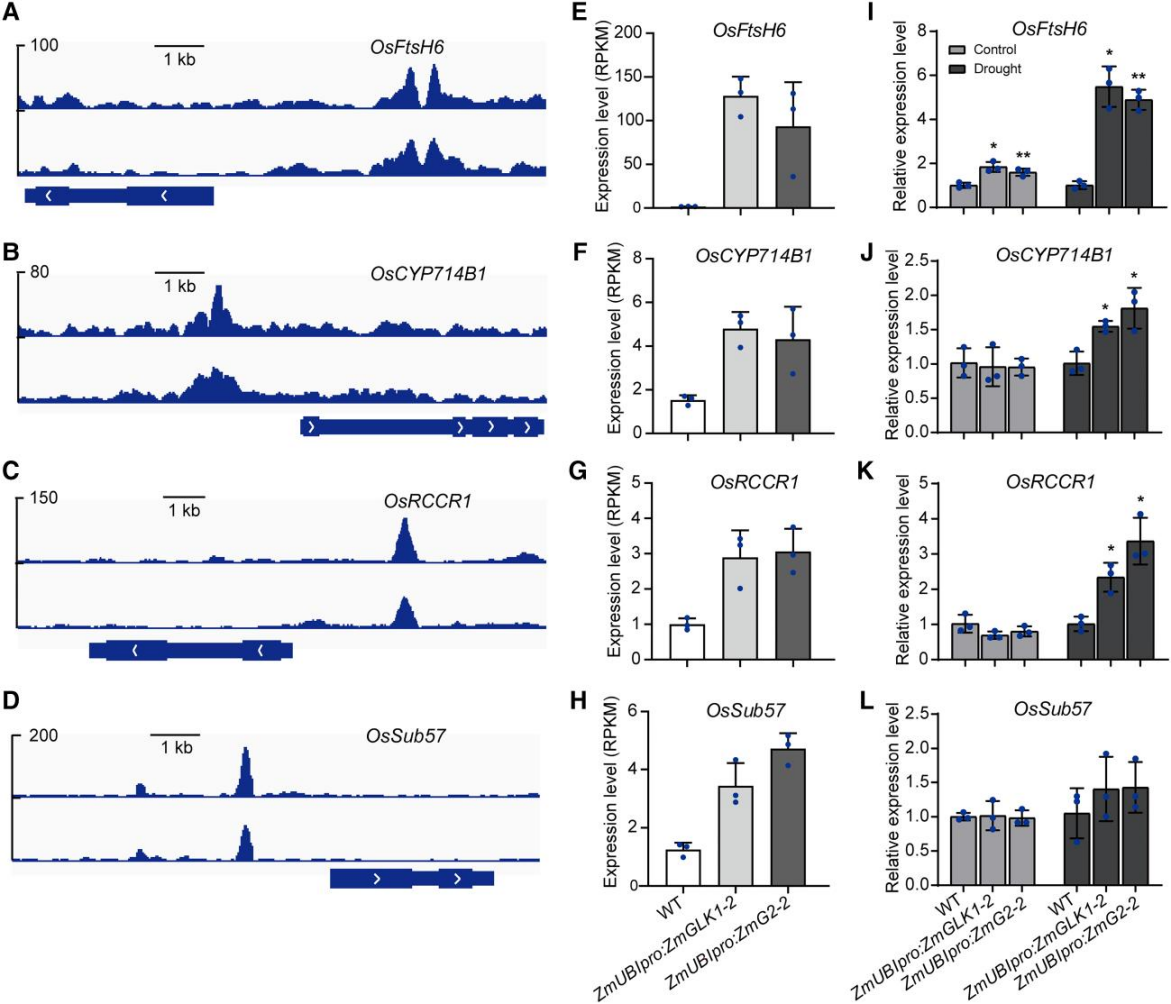
Putative ZmGLK1 and ZmG2 target genes in rice. **A to D)** DAP-seq indicated that ZmGLK1 and ZmG2 preferentially bound to the promoters of *OsSub57***A)**, *OsFtsH6***B)**, *OsCYP714B1***C)**, and *OsRCCR***D)**. **E to H)** Expression levels of *OsSub57***E)**, *OsFtsH6***F)**, *OsCYP714B1***G)**, and *OsRCCR***H)** in WT rice and in rice overexpressing *ZmGLK1* or *ZmG2* as determined with RNA-seq analysis. Gene expression was calculated in RPKM. **I to L)** Relative expression levels of *OsSub57***I)**, *OsFtsH6***J)**, *OsCYP714B1***K)**, and *OsRCCR***L)** in WT, *ZmUBIpro*:*ZmGLK1*, and *ZmUBIpro*:*ZmG2* rice under control conditions and after 7 d of drought stress as determined with RT-qPCR. Data are presented as the mean ± Sd from 3 biological replicates. *P < 0.05, **P < 0.01 (Student's *t* test).

A genome-wide transcriptomic analysis was also conducted in WT, *ZmUBI_pro_*:*ZmGLK1*, and *ZmUBI_pro_*:*ZmG2* rice plants at 3 h after ABA treatment to investigate the global effects of ZmGLK1 and ZmG2 introduced by ABA, especially on stomatal movement. WT plants clearly showed distinct expression patterns compared with *ZmUBI_pro_*:*ZmGLK1* and *ZmUBI_pro_*:*ZmG2* plants, as demonstrated by the clear separation with principal component analysis (PCA; Fig. 6A). Specifically, after ABA treatment, 702 and 775 genes were significantly upregulated in *ZmUBI_pro_*:*ZmGLK1* and *ZmUBI_pro_*:*ZmG2* plants, respectively, compared with the WT, of which 482 genes were upregulated in both transgenic lines (Fig. 6B). Gene Ontology (GO) term enrichment analysis revealed that the upregulated differentially expressed genes (DEGs) in *ZmUBI_pro_*:*ZmGLK1* and *ZmUBI_pro_*:*ZmG2* plants functioned in multiple biological processes but primarily in the ABA and water deprivation pathways (Fig. 6, C and D). Next, we performed DNA affinity purification sequencing (DAP-seq) analysis to identify genes directly regulated by the ZmGLK TFs. This analysis revealed 6,601 and 6,565 putative binding sites of ZmGLK1 and ZmG2 in the rice genome, respectively, with more than half of the identified sites being bound by both ZmGLK and ZmG2 (Supplemental Fig. S4A). Of the 3,835 binding sites shared by ZmGLK1 and ZmG2, 17.44% were localized to promoters, 8.59% to exons, and 45.26% to intergenic regions (Supplemental Fig. S4B). Motif analysis demonstrated that the most enriched core motifs found in the ZmGLK1- and ZmG2-binding regions were GCCTCT and AGATTCT (Supplemental Fig. S4, C and D). Fifty-nine genes identified from the DAP-seq data as potential targets of ZmGLK1 and ZmG2 in rice were also identified from the RNA-sequencing (RNA-seq) data as differentially expressed in plants overexpressing *ZmGLK1* or *ZmG2* (Fig. 6B; Supplemental Table S1). We noticed 4 upregulated DEGs were annotated to abiotic stress tolerance and showed strong binding peaks in the DAP-seq analysis simultaneously. Therefore, these genes were identified as putative target genes of ZmGLK1 and ZmG2 in rice, including rice genes *Filamentation Temperature Sensitive Protein H6* (*OsFtsH6*), *Cytochrome P450 Family 714 B1* (*OsCYP714B1*), *Red Chlorophyll Catabolite Reductase 1* (*OsRCCR1*), and *Subtilisin-like Protease 57* (*OsSub57*; Fig. 7, A to D). The gene expression from RNA-seq data of these 4 genes was prominently higher in *ZmUBI_pro_*:*ZmGLK1* and *ZmUBI_pro_*:*ZmG2* rice plants (Fig. 7, E to H). Further reverse transcription quantitative PCR (RT-qPCR) analysis verified that these genes were highly induced in *ZmUBI_pro_*:*ZmGLK1* and *ZmUBI_pro_*:*ZmG2* rice under drought stress conditions (Fig. 7, I to L). These putative target genes may contribute to enhanced drought tolerance by enabling rapid stomatal movement when suffering from water deficit.

## Discussion

GLK TFs have long been regarded as some of the most important regulators of chloroplast biogenesis and photosynthetic organelle formation; they have been identified in Arabidopsis, tomato (*Solanum lycopersicum* L.), and maize (Rossini et al. 2001; Waters et al. 2009; Powell et al. 2012). In rice, ectopic expression of maize *GLK* genes (*ZmGLK1* and *ZmG2*) promotes a proto-Kranz status in the leaf anatomy, increasing chloroplast and mitochondrial development in rice vascular sheath cells (Wang et al. 2017). A previous study by our lab has revealed that rice plants overexpressing maize *GLK* genes have increased biomass and grain yield as a result of improved photosynthetic capacity and reduced photoinhibition under high- and fluctuating-light conditions (Li et al. 2020).

In the present study, we uncovered that overexpression of maize *GLK* genes (*ZmGLK1* and *ZmG2*) in rice enhanced drought tolerance by promoting stomatal closure. Specifically, when plants were grown under standard, well-watered conditions, we observed smaller stomatal size but higher stomatal density and stomatal aperture in rice plants overexpressing *ZmGLK1* or *ZmG2* compared with WT plants (Fig. 2, B and E). These results were consistent with earlier studies showing that *ZmGLK1* and *ZmG2* overexpression led to increased stomatal conductance in field-grown rice (Li et al. 2020), greenhouse-grown rice (Yeh et al. 2022), and Arabidopsis (Nagatoshi et al. 2016). In contrast, under drought stress, the stomata of *ZmGLK1*- or *ZmG2*-overexpressing rice plants rapidly closed (Figs. 2B and 3E), improving drought tolerance by preventing water loss. Previous studies in rice have reported that small, high-density stomata can close quickly, thus promoting resilience against drought stress (Caine et al. 2019; Caine et al. 2023); these prior results were consistent with those of the present study. Notably, differences in stomatal status between control and drought-stressed plants as a result of *ZmGLK1* or *ZmG2* overexpression were directly caused by regulation of genes involved in stomatal movement, namely inward K^+^ channels and an H^+^-ATPase (e.g. *OsKATs*, *OsAKT1*, and *OsAHK7*; Fig. 5). Upregulation of K^+^_in_ channel genes by *ZmGLK1* or *ZmG2* overexpression under normal conditions was in line with a previous study in Arabidopsis showing that GLK is a positive regulator of K^+^ channel genes and stomatal movement (Nagatoshi et al. 2016); thus, this rapid stomatal closure of transgenic rice plants resulted directly from a significant reduction in the expression levels of those genes under drought conditions.

Notably, we verified that the regulation of rapid stomatal closure in response to water deficit was ABA mediated, supported by the exogenous application of ABA inducing faster stomatal closure in *ZmUBI_pro_*:*ZmGLK1* and *ZmUBI_pro_*:*ZmG2* lines compared with the WT (Fig. 4B), which mimicked the effects of drought stress. Our finding is consistent with the previous study that suggested the fast stomatal closure requires a high ABA sensitivity (Candido-Sobrinho et al. 2022). Our results also implied that ZmGLKs may function in the ABA biosynthesis pathway, as indicated by the higher ABA accumulation (Supplemental Fig. S5) along with the abundant expression of several key genes involved in ABA biosynthesis (e.g. *OsNCED2*, *OsNCED3*, *OsAAO3*, and *OsZEP1*) in response to drought (Supplemental Fig. S6). ABA biosynthesis starts with the epoxidation of zeaxanthin, and this xanthophyll precursor therefore plays an important role in ABA biosynthesis. We previously discovered that ZmGLKs increase levels of xanthophylls, including zeaxanthin and lutein (Li et al. 2020), which may lead to the improved ABA biosynthesis in that way. Moreover, a study in Arabidopsis showed that GLKs directly activate the expression of *WRKY40*, and GLK-WRKY40 together negatively regulates ABA signaling (Ahmad et al. 2019), suggesting a possible regulatory role of ZmGLKs in the ABA signaling pathway. We also proposed that the C_4_-like traits conferred by ZmGLKs as mentioned above may contribute to the rapid stomatal closure. This has been demonstrated by model simulations and experimental data that major C_4_ crops are capable of more rapid stomatal closure compared to C_3_ crops in response to water deficit, resulting in the high water use efficiency (WUE) (McAusland et al. 2016; Wang et al. 2021; Ozeki et al. 2022). Notably, previous studies have demonstrated that slower stomatal closure in ferns is associated with reduced responsiveness to ABA and sugars compared to angiosperms (Lima et al. 2019; Candido-Sobrinho et al. 2022), while the rapid transport of ions and osmolytes between guard cells and subsidiary cells in grass species contributes to the fast stomatal movement (Chen et al. 2017). Rice plants overexpressing *ZmGLK*s have improved carbohydrate contents (Li et al. 2020), consistent with *SlGLK* gene expression in tomato plants (Powell et al. 2012; Nguyen et al. 2014); this may contribute to rapid stomatal closure at the metabolic level.

To further reveal the mechanism underlying ZmGLK-regulated stomatal movement, we conducted a comparative analysis of RNA-seq and DAP-seq data. This analysis revealed several potential target genes showing strong binding peaks, including *OsFtsH6*, *OsCYP714B1*, *OsRCCR1*, and *OsSub57* (Fig. 7). *OsFtsH6*, which belongs to the *OsFtsH* gene family, is involved in D1 turnover as part of the PSII repair cycle. D1 turnover comprises removal of damaged D1 proteins by FtsH proteases located in the chloroplast, followed by coordinated assembly of newly synthesized D1 proteins into the thylakoid membrane (Wang et al. 2016). The high levels of D1 protein observed in *ZmGLK1*- and *ZmG2*-overexpressing plants in our previous study (Li et al. 2020) prompted us to hypothesize the potential regulatory function of ZmGLKs on *OsFtsH6* expression. *OsCYP714B1* encodes a gibberellin (GA) 13-oxidase that plays a critical role in GA 13-hydroxylation to regulate plant growth (Magome et al. 2013). *OsRCCR1* encodes a chlorophyll degradation enzyme; knocking down *OsRCCR1* leads to chlorotic lesions in older leaves and early senescence (Tang et al. 2011). Further, *OsSub57* is annotated as encoding a subtilisin homolog that is salt and drought induced (Landi et al. 2017; Zheng et al. 2022), but its function remains unknown. Nevertheless, it remains an open question whether the transcriptional regulation conferred by the heterologous gene is conserved or distinct from the native species, due to the complexity of gene regulatory system.

Stomatal closure is considered as the first reaction to drought stress in most plants, preventing water loss through transpiration. Improving the rapidity of stomatal response is a feasible and effective strategy to maximize photosynthesis and WUE simultaneously (Lawson and Vialet-Chabrand 2019). It is worth noting that investigations into the functions of the potential target genes and regulatory roles as well as the quantification of stomatal kinetics of *ZmGLK* overexpression plants are still needed to understand the mechanism by which ZmGLKs fine-tune stomatal movements, to coordinate trade-offs between photosynthesis and drought tolerance. Further exploration will provide insights and useful targets for crop breeding, enabling creation of elite varieties with both high photosynthetic capacity and drought tolerance.

## Materials and methods

### Plant growth conditions

The WT rice (*O. sativa* spp. *japonica*) cultivar ‘Kitaake’ and 2 homozygous lines described by Li *et al*. (2020) (*ZmUBI_pro_*:*ZmGLK1* and *ZmUBI_pro_*:*ZmG2*) were used in this study. For hydroponic culture, rice seedlings were grown in modified Kimura B solution (0.5 mM (NH_4_)_2_SO_4_, 0.54 mM MgSO_4_·7H_2_O, 1 mM KNO_3_, 0.3 mM CaCl_2_, 0.18 mM KH_2_PO_4_, 0.09 mM K_2_SO_4_, 16 *µ*M Na_2_SiO_3_·9H_2_O, 9.14 *µ*M MnCl_2_·4H_2_O, 46.2 *µ*M Na_2_MoO_4_·2H_2_O, 0.76 *µ*M ZnSO_4_·7H_2_O, 0.32 *µ*M CuSO_4_·5H_2_O, and 40 *µ*M Fe(II)-EDTA, pH 5.8) in a growth chamber at 28 °C under a 16-h/8-h light/dark cycle with 200 to 300 *µ*mol/m^2^/s photon intensity. Nutrient solution was renewed every 3 d.

### Drought stress treatments

For drought stress treatments at the seedling stage, germinated rice seeds were planted in 12 × 15 cm pots filled with 250 g of paddy soil and grown in a growth chamber under the conditions described above. All pots were irrigated with same amount of water for 3 wk. Then, the seedlings were gradually drought stressed by withholding water for 10 d, followed by rewatering for 6 d. The control group was irrigated normally for the duration of the experiment.

For drought treatment at the tilling stage, germinated rice seeds were planted in 5-L pots filled with 3 kg of paddy soil. The pots were put in a water container and grown in the greenhouse with natural sunlight. After 60 d, plants were subjected to drought stress by stopping water supply for 10 d, with soil water content monitored using the soil moisture meter. After rewatering for 7 d, the survival rate was measured by counting the number of plants with at least 1 fully expanded leaf. Transgenic lines and WT were planted in the same pots with at least 3 replicates for each treatment.

To evaluate the sensitivity of rice seedling to osmotic stress, 3-wk-old hydroponically grown rice seedlings were transferred to nutrient solution containing 20% (w/v) PEG 6000 for 10 d to simulate drought stress. Seedlings grown in the normal nutrient solution served as the control. All hydroponic solutions were renewed every 3 d.

### Gas exchange and chlorophyll fluorescence measurements

Gas exchange parameters were measured with a LI-COR 6400XT instrument (LI-COR Biosciences, USA) in the topmost fully expanded leaves of rice seedlings using a 2 × 3 cm leaf chamber and a red–blue light source. The PPFD was 1,200 *µ*mol/m^2^/s, and the CO_2_ concentration within the chamber was 400 *µ*mol/mol. Leaf chlorophyll fluorescence parameter *F*_v_/*F*_m_ indicates the maximum quantum efficiency of PSII was measured with a FluorPen FP100 (PSI, Czech Republic) after dark adaptation for 15 to 20 min.

### Stomatal trait measurements with scanning electron microscopy

Rice leaves were detached from control or drought-treated plants and immediately cut into 3 × 3 mm pieces, excluding the veins. Samples were directly fixed in 2.5% (v/v) glutaraldehyde in 0.1 M phosphate buffer (pH 7.0) and then fixed with 1% osmium tetroxide. After washing twice with 0.1 M phosphate buffer, samples were dehydrated gradually in an ethanol series (30%, 50%, 60%, 70%, 80%, 90%, and 100%) for 15 min each, followed by incubating in tertiary butanol for 35 min. Then, samples were dried using a critical point dryer, pasted on the sample stage, and then coated with gold. Stomata were observed and photographed using a SU-8010 scanning electron microscope (Hitachi, Japan). The size, number, and aperture sizes of stomata were calculated using ImageJ software.

### Quantification of endogenous ABA content

The uppermost expanded leaves of control and drought-stressed rice seedlings were detached and flash frozen in liquid nitrogen. Ground samples (100 mg each) were extracted with an acetonitrile solution containing an internal standard at 4 °C overnight. Samples were centrifuged, and the resulting supernatant was extracted again. The combined extracts were purified on a C_18_ silica column and dried with nitrogen gas. After resolving in methanol and passing through a 0.22-µm filter, ABA was quantified on a HPLC–tandem mass spectroscopy (MS/MS) system as described by Liu et al. (2012).

### Exogenous ABA treatment

Forty-day-old rice seedlings grown in pots were sprayed with 100 *µ*M ABA solution (containing 0.5% [v/v] Tween-20 as a surfactant) until the leaves were moist. The volume of ABA solution applied was consistent between seedlings. At 2.5 h after treatment, gas exchange parameters and stomatal traits were evaluated as described above.

### RNA extraction and RT-qPCR

The uppermost fully expanded leaves were harvested from 3-wk-old rice seedlings grown in pots under normal conditions or drought stress for 7 d. Samples were flash frozen in liquid nitrogen and ground to powder, and then total RNA was extracted with TRIzol reagent (Invitrogen). RNA purity and quantity were evaluated using a NanoDrop 2000 spectrophotometer (Thermo Fisher Scientific, USA). After DNase treatment, cDNA was synthesized from 1 *µ*g of total RNA per sample using the RevertAid First Strand cDNA Synthesis Kit (Thermo Fisher Scientific, USA). RT-qPCR was performed using KOD SYBR Green mix with ROX (TOYOBO) on an ABI QuantStudio 6 Flex instrument (Applied Biosystems, USA). Relative transcript levels were calculated with the 2^−ΔΔCT^ method (Livak and Schmittgen 2001) with 3 biological replicates for each treatment, using *OsActin* as the internal control. Primers are listed in Supplemental Table S2.

### RNA-seq analysis

At 3 h after exogenous ABA treatment, leaves were collected from 4-wk-old rice seedlings grown in pots. Total RNA was extracted with TRIzol reagent, and then RNA integrity was assessed with the Agilent 2100 Bioanalyzer (Agilent Technologies, USA). RNA-seq libraries were constructed from WT, *ZmUBI_pro_*:*ZmGLK1-3*, and *ZmUBI_pro_*:*ZmG2-3* rice plants using the TruSeq Stranded mRNA LT Sample Prep Kit (Illumina, USA) with 3 biological replicates per line. The resulting 9 libraries were sequenced on the Illumina HiSeq X Ten sequencing platform. After removing the adaptor sequences and low-quality reads, clean reads were mapped to the *O. sativa* cv. ‘Nipponbare’ reference genome using HISAT (Kim et al. 2015) and Bowtie2 (Langmead et al. 2009). Gene expression levels were calculated in reads per kilobase of transcript per million mapped reads (RPKM) using Cufflinks. DEGs were identified with the “DESeq” R package. The thresholds for classification as a DEG in the transgenic lines compared to the WT were *P* < 0.05 and |log_2_(fold change)| >1.

### DAP-seq and data analysis

The full-length coding sequences of *ZmGLK1* and *ZmG2* were amplified from cDNA of the maize accession B73. Each sequence was recombined into the pIX-HALO vector using LR Clonase II (Invitrogen). The HALO-ZmGLK1 and HALO-ZmG2 proteins were generated using 500 ng each of the pIX-HALO-ZmGLK1 and pIX-HALO-ZmG2 plasmids and the TNT SP6 Coupled Reticulocyte Lysate System (Promega) following the manufacturer's protocol. The resulting proteins were immediately incubated with 10 *µ*L of Magne-HALOTag beads (Promega) for 1 h at 25 °C in 1× phosphate-buffered saline (PBS) with 0.005% (v/v) Nonidet P-40 and 0.1% (v/v) Tween. Bound proteins were washed 5 times with PBS with 0.1% Tween (PBST) and then digested with DNaseI. The DNA-free HALO-ZmGLK1 and HALO-ZmG2 proteins were then incubated with 500 *μ*g of ultrasonically fragmented ‘Nipponbare’ genomic DNA (200- to 800-bp fragments) for 1 h at 25 °C. The beads were washed 8 times with PBST, then elution buffer was added, and the beads were incubated at 98 °C for 10 min. Sequencing libraries were constructed from the eluted DNA using the TruePrep DNA Library Prep Kit V2 for Illumina (Vazyme). The raw sequencing data were cleaned as described above for the RNA-seq data. Clean reads were mapped to the ‘Nipponbare’ reference genome (MSU TIGR v7) using Bowtie2. Peak calling was performed using MACS2. Peaks were visualized from the BigWig files using the integrated genomics viewer. HOMER (with default parameters) was used to identify motifs in the 150-bp regions both upstream and downstream of peaks.

### Statistical analysis

Statistically significant differences between WT and *ZmUBI_pro_*:*ZmGLK1* or *ZmUBI_pro_*:*ZmG2* were assessed for all experiments with Student's *t* test in Microsoft Excel. Differences were considered significant at *P* < 0.05. Figures were generated with GraphPad Prism 9.0 and Adobe Illustrator CS3.

### Accession numbers

Raw sequence data generated in this study have been deposited in the NCBI BioProject database under accession number PRJNA1018861 for RNA-seq and PRJNA1019016 for DAP-seq. The sequence data from this article can be found in the GenBank/EMBL data libraries under the following accession numbers: *ZmGLK1* (GenBank: AF318580) and *ZmG2* (GenBank: AF318579).

## Supplementary Material

kiad561_Supplementary_Data

## Data Availability

The data underlying this article are available in the article and in its online supplementary material.

## Supplemental data

The following materials are available in the online version of this article.

**Supplemental Figure S1.** Enhanced tolerance of *ZmUBIpro*:*ZmGLK1* and *ZmUBIpro*:*ZmG2* rice plants to drought stress induced by 20% PEG 6000.

**Supplemental Figure S2.** Overexpression of *ZmGLK1* or *ZmG2* in rice led to decreased stomatal conductance and photosynthetic parameters in response to drought.

**Supplemental Figure S3.** Dynamic changes of soil water content during the drought stress in the greenhouse experiment.

**Supplemental Figure S4.** Genome-wide summary of the regulatory network downstream of ZmGLK1 and ZmG2 based on DAP-seq data.

**Supplemental Figure S5.** Changes in endogenous ABA content in WT, *ZmUBI_pro_*:*ZmGLK1*, and *ZmUBI_pro_*:*ZmG2* rice leaves under normal conditions and after 7 d of drought stress.

**Supplemental Figure S6.** Relative expression levels of ABA biosynthesis genes in the leaves of WT, *ZmUBI_pro_*:*ZmGLK1*, and *ZmUBI_pro_*:*ZmG2* rice plants under normal conditions and after 7 d of drought stress.

**Supplemental Table S1.** Relative change of gene expression level of 59 overlapped genes from RNA-seq and DAP-seq analyses.

**Supplemental Table S2.** Primers used for RT-qPCR.

## Dive Curated Terms

The following phenotypic, genotypic, and functional terms are of significance to the work described in this paper:

AAO3 Gramene: AT2G27150
AAO3 Araport: AT2G27150

WRKY40 Gramene: MDP0000177906
WRKY40 Araport: MDP0000177906

OsLEA3 Gramene: LOC_Os05g46480
OsLEA3 Araport: LOC_Os05g46480

OsNCED3 Gramene: LOC_Os03g44380
OsNCED3 Araport: LOC_Os03g44380

OsbZIP23 Gramene: LOC_Os02g52780
OsbZIP23 Araport: LOC_Os02g52780
