# Correction to: Maize GOLDEN2-LIKE proteins enhance drought tolerance in rice by promoting stomatal closure

**DOI:** 10.1093/plphys/kiad649

**Published:** 2023-12-11

This is a correction to: Xia Li, Jing Li, Shaobo Wei, Yuan Gao, Hongcui Pei, Rudan Geng, Zefu Lu, Peng Wang, Wenbin Zhou, Maize GOLDEN2-LIKE proteins enhance drought tolerance in rice by promoting stomatal closure, *Plant Physiology*, 2023, kiad561, https://doi.org/10.1093/plphys/kiad561

In the originally published version of this manuscript, Fig. 2B showed the stomatal number within 0.12 mm^2^, which was the original area from the micoscopy image. The authors missed the data conversion process from 0.12 mm^2^ to mm^2^.

The authors have recalculated the stomatal number, and the corrected Fig. 2 is included here.

**Figure 2:**
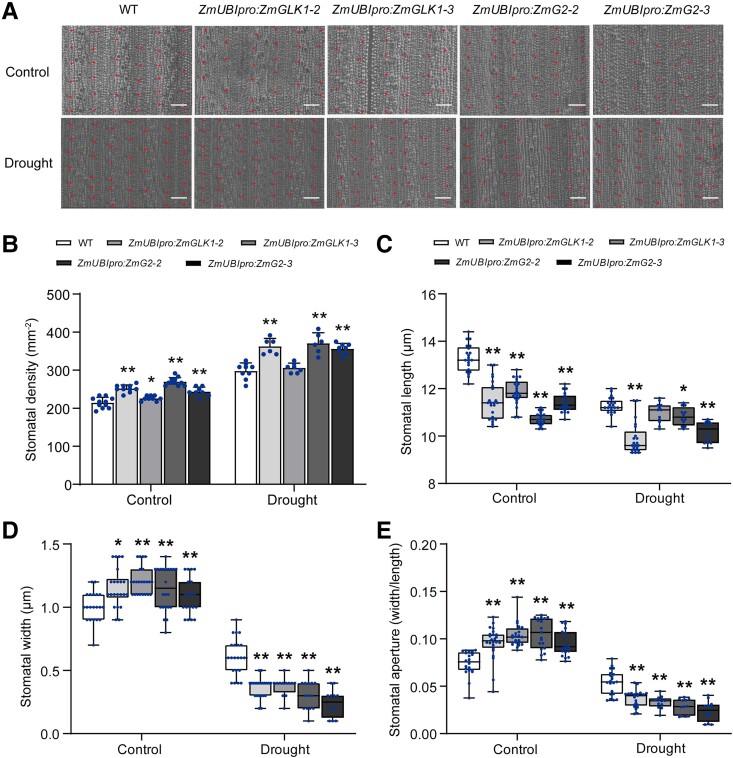


This error has been corrected online.

