# Supplementary material for: Maize GOLDEN2-LIKE proteins enhance drought tolerance in rice by promoting stomatal closure
Source: Plant Physiol. 2023 Oct 18;194(2):774–86. doi: 10.1093/plphys/kiad561 (PMC10828204; doi:10.1093/plphys/kiad561)
Supplement: kiad561_Supplementary_Data [file kiad561_supplementary_data.pdf]

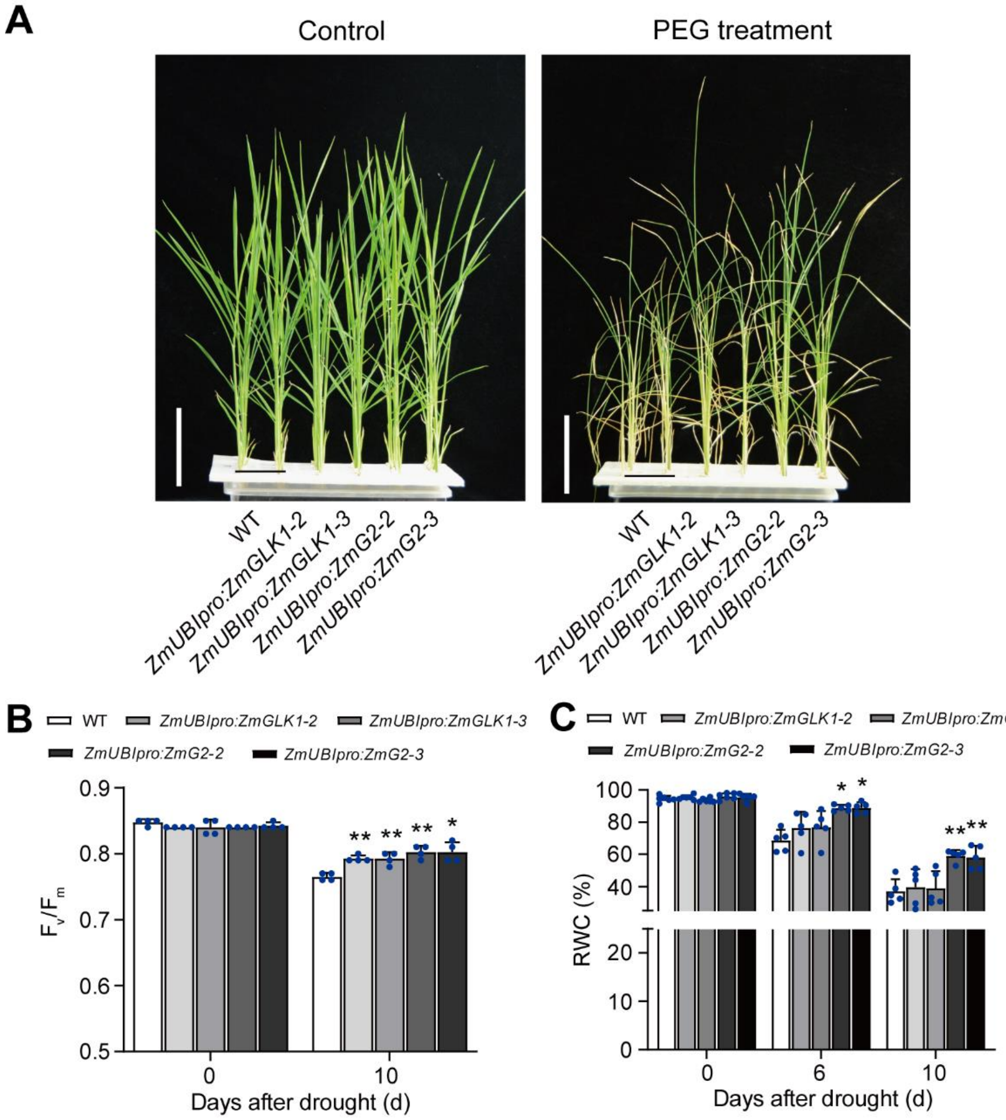

Supplemental Figure S1. Enhanced tolerance of *ZmUBIpro*:*ZmGLK1* and *ZmUBIpro*:*ZmG2* rice plants to drought stress induced by 20% polyethylene glycol (PEG) 6000.

(**A**) Representative images showing phenotypes of control four-week-old wild-type (WT), *ZmUBI*$_{pro}$:*ZmGLK1*, and *ZmUBI*$_{pro}$:*ZmG2* rice seedlings (left) and plants treated with 20% PEG 6000 for 10 d (right). Scale bar: 5 cm. (**B**) Relative water content (RWC) and (**C**) F_v/F_m values of control WT, *ZmUBI*$_{pro}$:*ZmGLK1*, and *ZmUBI*$_{pro}$:*ZmG2* rice seedlings and plants treated with 20% PEG 6000 for 10 d. Data are presented as the mean ± standard deviation from four to six biological replicates. *$p < 0.05$, **$p < 0.01$ (Student's *t*-test).

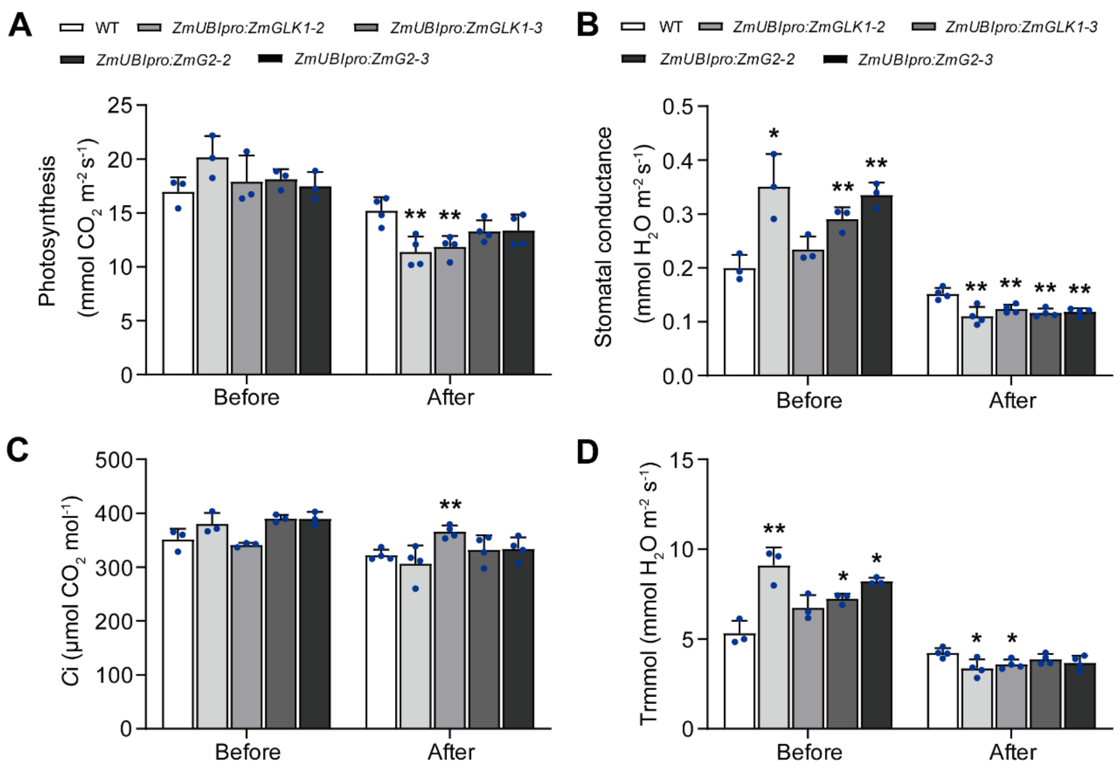

Supplemental Figure S2. Overexpression of *ZmGLK1* or *ZmG2* in rice led to decreased stomatal conductance and photosynthetic parameters in response to drought.

(**A**) Photosynthesis rates, (**B**) stomatal conductance, (**C**) intercellular CO_2 concentrations (*C*i), and (**D**) transpiration rates in three-week-old wild-type (WT), $ZmUBI_{pro}$:*ZmGLK1*, and $ZmUBI_{pro}$:*ZmG2* rice plants grown in soil under normal growth conditions or drought stress for 7 d. Data are presented as the mean ± standard deviation from three biological replicates. *$p < 0.05$, **$p < 0.01$ (Student's *t*-test).

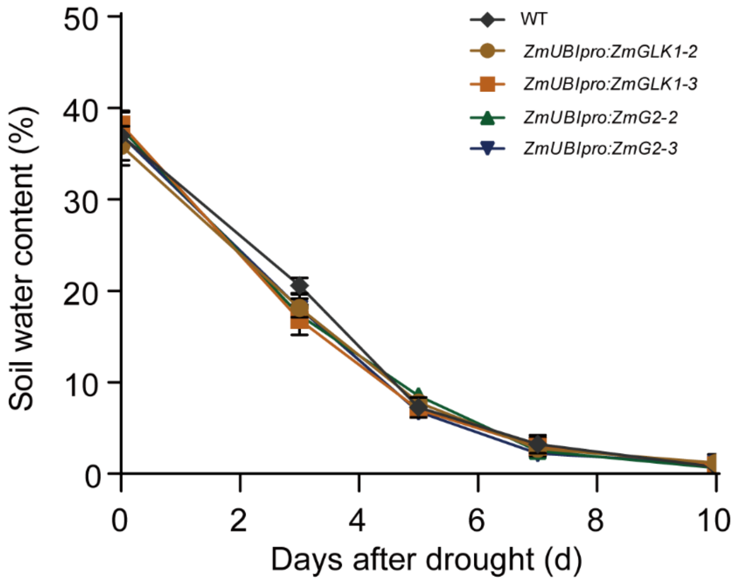

Supplemental Figure S3. Dynamic changes of soil water content during the drought stress in the greenhouse experiment.

Soil water contents were measured by soil moisture sensors after 0, 3, 5, 7, 10 days of drought treatment. Data are presented as the mean ± standard deviation from three independent pots.

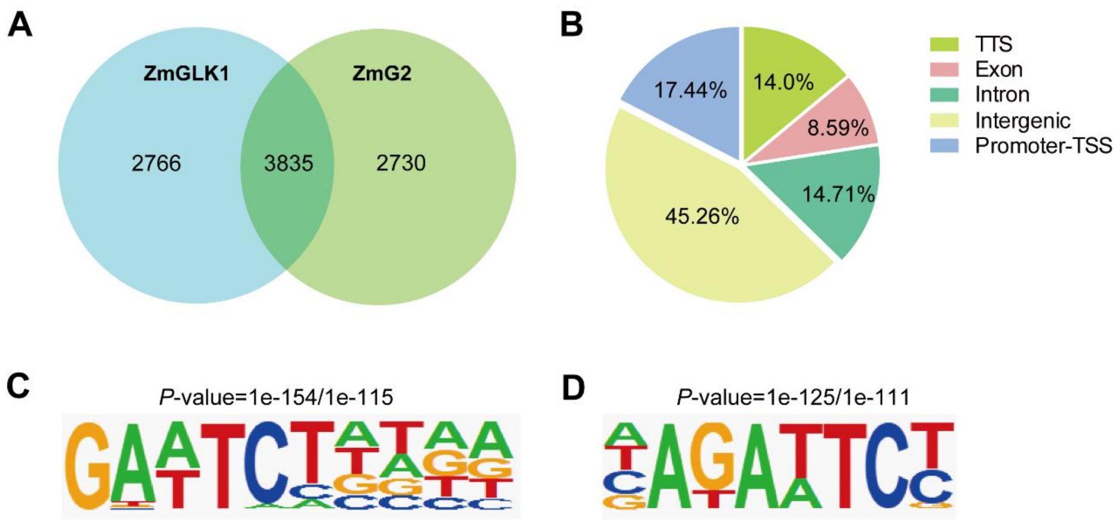

Supplemental Figure S4. Genome-wide summary of the regulatory network downstream of ZmGLK1 and ZmG2 based on DNA-affinity purification sequencing (DAP-seq) data.

(**A**) Unique and overlapping genes determined from DAP-seq analysis to be targets of ZmGLK1 and ZmG2 in rice. (**B**) Distribution of the 3,835 binding regions shared by ZmGLK1 and ZmG2 in the rice genome. TSS, transcription start site; TTS, transcription termination site. (**C-D**) The two most strongly enriched motifs within the ZmGLK1 (**C**) and ZmG2 (**D**) binding peaks. *p*-values were calculated in the MEME suite of tools.

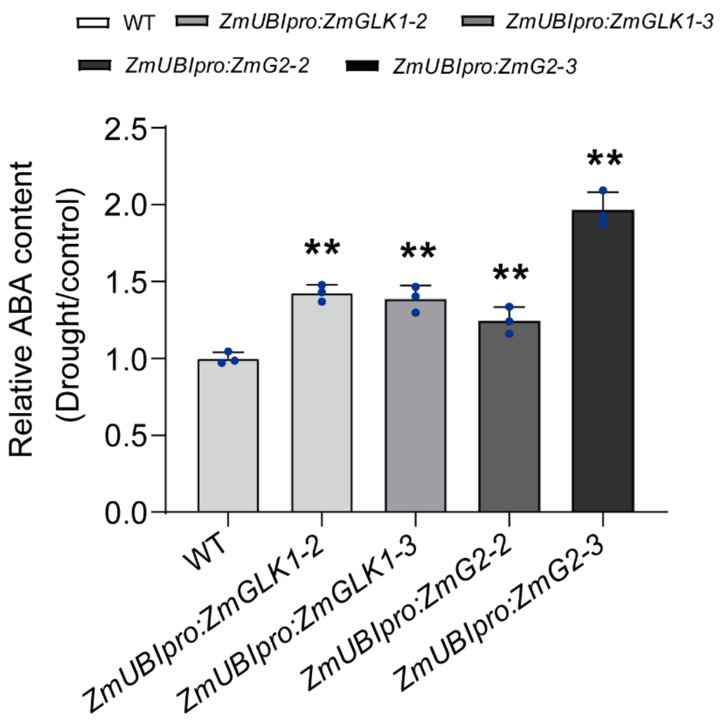

Supplemental Figure S5. Changes in endogenous abscisic acid (ABA) content in WT, *ZmUBI$_{pro}$:ZmGLK1*, and *ZmUBI$_{pro}$:ZmG2* rice leaves under normal conditions and after 7 d of drought stress.

Relative ABA content was calculated as the ratio of ABA content after drought stress for 7 d to the ABA content under control conditions. Data are presented as the mean ± standard deviation from three biological replicates. ***p* < 0.01 (Student's *t*-test).

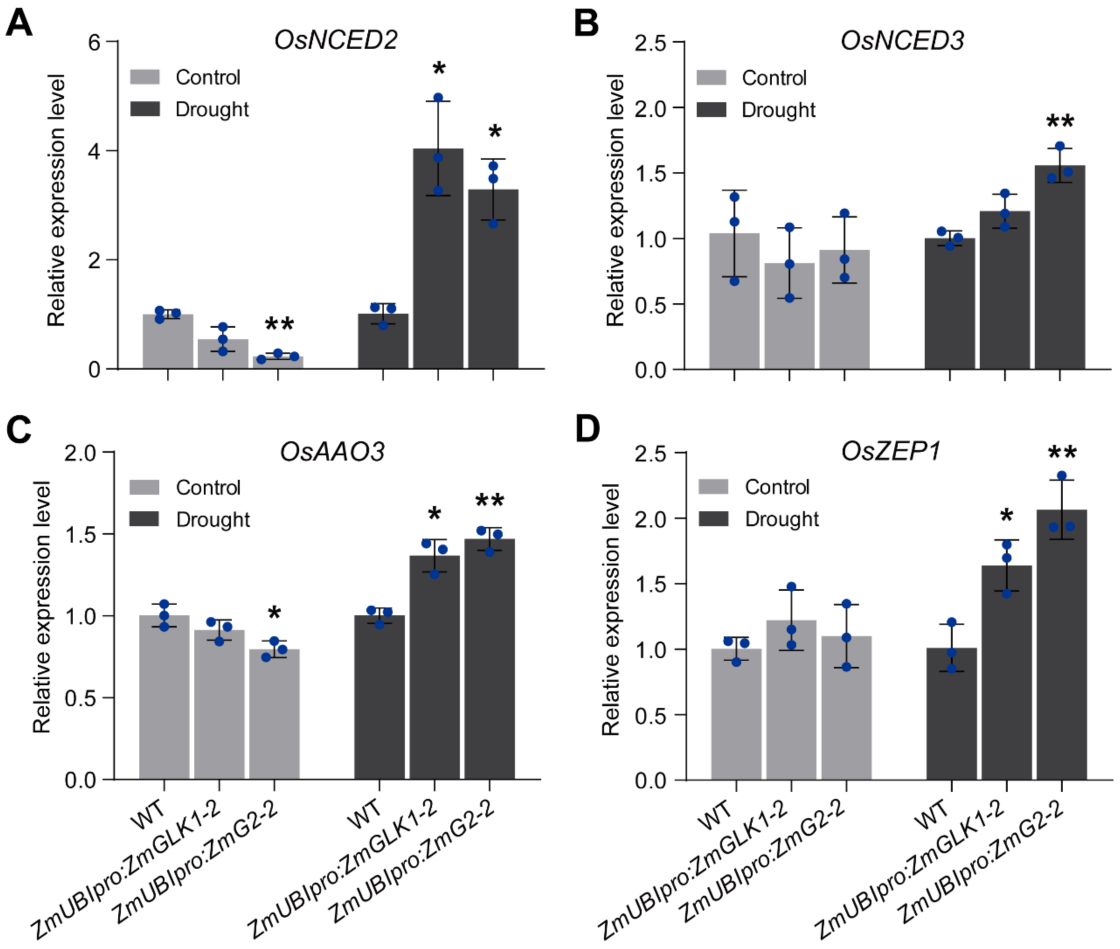

Supplemental Figure S6. Relative expression levels of ABA biosynthesis genes in the leaves of WT, *ZmUBI*$_{pro}$*:ZmGLK1*, and *ZmUBI*$_{pro}$*:ZmG2* rice plants under normal conditions and after 7 d of drought stress.

Relative expression levels of (**A**) *OsNCED2*, (**B**) *OsNCED3*, (**C**) *OsAAO3*, and (**D**) *OsZEP1* in three-week-old rice plants grown in soil under normal conditions or after 7 d of drought stress. Gene expression was measured with reverse transcription quantitative PCR (RT-qPCR). Data are presented as the mean ± standard deviation from three biological replicates. *$p < 0.05$, **$p < 0.01$ (Student's *t*-test).

Supplemental Table S1. Relative change of gene expression level of 59 overlapped genes from RNA-seq and DAP-seq analysis.

Gene ID	Log$_2$FC		Putative function
	***ZmGLK1*-OE1/WT**	***ZmG2*-OE1/WT**	
**LOC_Os06g12370**	6.602	6.136	OsFtsH6 - FtsH protease, homologue of AtFtsH6, expressed
LOC_Os01g10370	4.316	4.349	AP2 domain containing protein, expressed
LOC_Os06g46160	3.727	3.511	expressed protein
LOC_Os02g04780	3.461	3.17	expressed protein
LOC_Os05g05930	3.415	2.296	peripheral-type benzodiazepine receptor, putative, expressed
LOC_Os10g05490	3.331	2.476	cytochrome P450, putative, expressed
LOC_Os12g27254	3.103	4.322	transferase family protein, putative, expressed
LOC_Os03g17790	2.806	2.649	OsRCI2-5 - Putative low temperature and salt responsive protein, expressed
LOC_Os12g28590	2.796	3.464	ATPase 2, putative, expressed
LOC_Os08g41960	2.76	2.146	OsMADS37 - MADS-box family gene with MIKC* type-box, expressed
LOC_Os08g40910	2.74	2.346	expressed protein
LOC_Os05g01140	2.393	3.001	methyltransferase, putative, expressed
LOC_Os07g12340	2.37	2.157	NAC domain-containing protein 67, putative, expressed
LOC_Os04g06590	2.35	2.491	expressed protein
LOC_Os01g43851	2.123	2.293	cytochrome P450 72A1, putative, expressed
LOC_Os03g08090	2.109	1.955	hydrolase, alpha/beta fold family protein, putative, expressed
LOC_Os10g22310	2.082	2.309	glutathione S-transferase GST 26, putative, expressed
LOC_Os06g35960	2.08	2.164	HSF-type DNA-binding domain containing protein, expressed

LOC_Os02g52170	2.03	2.43	expressed protein
LOC_Os11g26570	2.007	2.059	dehydrin, putative, expressed
LOC_Os01g29330	1.987	1.286	expressed protein
LOC_Os02g30714	1.954	1.91	dehydrogenase/reductase, putative, expressed
LOC_Os10g24004	1.871	1.395	expressed protein
LOC_Os01g18170	1.859	3.221	Cupin domain containing protein, expressed
LOC_Os06g35050	1.807	2.077	arogenate dehydrogenase 1, chloroplast precursor, putative, expressed
LOC_Os01g74300	1.79	1.323	metallothionein, putative, expressed
LOC_Os09g02250	1.739	2.085	transposon protein, putative, unclassified, expressed
LOC_Os02g44090	1.738	1.3	zinc finger protein, putative, expressed
LOC_Os11g07980	1.639	1.691	ion channel nompc, putative, expressed
LOC_Os11g02520	1.618	2.741	WRKY104, expressed
LOC_Os02g45710	1.603	1.446	zinc finger, C3HC4 type domain containing protein, expressed
LOC_Os11g37200	1.594	1.319	transmembrane BAX inhibitor motif-containing protein, putative, expressed
LOC_Os03g49350	1.575	1.225	lipozygenase protein, putative, expressed
LOC_Os02g06779	1.519	1.717	expressed protein
**LOC_Os10g25030**	1.47	1.512	OsRCCR1 - red chlorophyll catabolite reductase, putative, expressed
**LOC_Os07g48330**	1.457	1.387	OsCYP714B1 - cytochrome P450, putative, expressed
LOC_Os02g26210	1.432	1.414	flowering promoting factor-like 1, putative, expressed
LOC_Os08g36680	1.397	1.894	bifunctional monodehydroascorbate reductase and carbonic anhydrasenectarin-3 precursor, expressed
**LOC_Os09g26920**	1.395	1.884	OsSub57 - Putative Subtilisin homologue, expressed
LOC_Os04g28420	1.394	1.534	peptidyl-prolyl isomerase, putative, expressed
LOC_Os06g18670	1.39	1.018	anthocyanidin 3-O-glucosyltransferase, putative, expressed

LOC_Os06g45710	1.375	1.388	phosphoglycerate kinase protein, putative, expressed
LOC_Os08g14320	1.332	1.166	zinc finger, C3HC4 type domain containing protein, expressed
LOC_Os07g43740	1.326	1.559	zinc finger, C3HC4 type domain containing protein, expressed
LOC_Os01g48446	1.28	1.254	no apical meristem protein, putative, expressed
LOC_Os06g14370	1.27	1.453	caleosin related protein, putative, expressed
LOC_Os09g20820	1.269	1.032	enolase, putative, expressed
LOC_Os08g32980	1.259	1.053	expressed protein
LOC_Os01g40260	1.25	1.689	WRKY77, expressed
LOC_Os05g45090	1.219	1.559	anthocyanidin 5,3-O-glucosyltransferase, putative, expressed
LOC_Os08g37874	1.218	1.143	2-nitropropane dioxygenase family, putative, expressed
LOC_Os12g13910	1.213	1.362	expressed protein
LOC_Os12g26290	1.183	2.075	alpha-DOX2, putative, expressed
LOC_Os06g13320	1.17	1.128	protein kinase, putative, expressed
LOC_Os11g26760	1.135	1.088	dehydrin, putative, expressed
LOC_Os02g47190	1.122	1.234	MYB family transcription factor, putative, expressed
LOC_Os08g04630	1.092	1.511	external NADH-ubiquinone oxidoreductase 1, mitochondrial precursor, putative, expressed
LOC_Os11g43990	1.051	1.035	expressed protein
LOC_Os05g34830	1.007	1.1	No apical meristem protein, putative, expressed

Note: Four potential target genes were marked in bold.

Supplemental Table S2. Primers used for RT-qPCR.

Name	Primer (5'-3')
OsKAT1-F	GTGCATCAACTACCTTATCGC
OsKAT1-R	CCAGAGGTTGAAGAGCATGTAG
OsKAT2-F	AAACTTTTCGCCATAGCTTGTC
OsKAT2-R	GTTGCTGATTTCAAGCATCCTT
OsKAT3-F	GTCCTAGTTGTTTACTCTGCCT
OsKAT3-R	TTTGCTATCGACATAAGCAACG
OsAKT1-F	AACTTCTTAAGCGTGGTATGGA
OsAKT1-R	ACTTTTCCTTCTGAGTCTCTGG
OsAHA7-F	TTCCATCACCATCCGTATAGTG
OsAHA7-R	AGAAGGCTTAACTCTGTCCTTC
OsZIP23-F	GGAGCAGCAAAAGAATGAGG
OsZIP23-R	GGTCTTCAGCTTCACCATCC
OsP5CS1-F	GCTGACATGGATATGGCAAAAC
OsP5CS1-R	GTAAGGTCTCCATTGCATTGCA
OsLEA3-F	TGAAGAGCACGGTGGTCGG
OsLEA3-R	GGCAGAGGTGTCCTTGTTGG
OsERD1-F	TCAAAGGGAAGACGAAGCATGG
OsERD1-R	GGGACGGAATACAACCATCTCA
OsNCED2-F	CAAACAGGCCGAACTTCTCG
OsNCED2-R	TGACCATTCTAAATCAAATGCTGGA
OsNCED3-F	CTCACATACAGCGGCAGCAC
OsNCED3-R	CGCTCGAGGACATTCGCCAC
OsAAO3-F	TGGAAGAGGATGTAGCCCGA
OsAAO3-R	GGCCTGTAAAGCCAGCATTG
OsZEP1-F	GGATGCCATTGAGTTTGGTT
OsZEP1-R	TGGCTGACTGAAGTCTCTCG
OsSub57-F	GGAACAGTGGTCCTTCTCCG
OsSub57-R	TTGTTGTACACGACCGGGAG
OsFtsH6-F	GCAGAAGGTGACGCTGAT
OsFtsH6-R	GAAGATCTGCTGCCTCGAC
OsCYP714B1-F	CCCCGACAAGGTCAAGGG
OsCYP714B1-R	CCCATGAACTGACTAGGACCTGT
OsRCCR1-F	GGATCGACGATTGATTTCATG
OsRCCR1-R	GTCGAGGCGTTCAGAAAGAT
OsActin-F	GGCACCACACCTTCTACAAT
OsActin-R	CTCACACCATCACCAGAGT
